# Books and Pamphlets Received

**Published:** 1887-04

**Authors:** 


### BOOKS AND PAMPHLETS RECEIVED.

*A Text-Book on Surgery—General, Operative and Mechanical.* By John A. Wyeth, M. D., Professor of Surgery in the New York Polyclinic ; Surgeon to the Mount Sinai Hospital ; Consulting Surgeon to the Yorkville Dispensary and Hospital for Women and Children ; to the Woman's Hospital of Brooklyn ; ex-President of the New York Pathological Society ; Member of the New York Surgical Society ; of the Academy of Medicine ; of the New York State Medical Association ; of the New York County Medical Society ; Honorary Member of the Texas State Medical Association ; of the College of Physicians and Surgeons of Little Rock, Ark. ; Author of an Essay on the Surgical Anatomy of the Tibio-tarsal Region, with special reference to Amputations of this Joint, awarded the James R. Wood Annual Prize of the Bellevue Alumni Association, 1876 ; An Essay on the Surgical Anatomy and History of the Carotid Arteries, awarded the First Prize of the American Medical Association, 1878 ; An Essay on the Surgical Anatomy and History of the Innominate and Subclavian Arteries, awarded the Second Prize of the American Medical Association, 1878. New York : D. Appleton & Co. 1887.

We have here a very recent surgical work of 777 very large pages, plain, bold type, neatly and elegantly printed, and substantially bound in superior white leather, and numerous bold, beautiful and admirably executed illustrations, plain and colored. The eminent Surgeon, Dr. Wyeth, has here presented a most valuable production. Though styled a text-book, it is admirably adapted as a work of reference for the surgeon and practitioner, giving, as it does, the recent and advanced views upon all surgical procedures. His remarks upon Bandaging, Anæesthesia, and Inflammation we have examined with special interest. The illustrations of operative procedures, especially amputations, are admirable ; so of the position and ligation of arteries. Fractures also, and plastic operations are well illustrated and ably described, and those relating to hernia are beautiful. In short, the entire book evinces the work of a master mind and a superior operator in surgery.

